# Mechanisms and pathways of *Toxoplasma gondii* transepithelial migration

**DOI:** 10.1080/21688370.2016.1273865

**Published:** 2016-12-20

**Authors:** Emily J. Jones, Tamas Korcsmaros, Simon R. Carding

**Affiliations:** a Gut Health and Food Safety Institute Strategic Programme, Institute of Food Research, Norwich Research Park, Norwich, UK; b Earlham Institute, Norwich Research Park, Norwich, UK; c Norwich Medical School, University of East Anglia, Norwich Research Park, Norwich, UK

**Keywords:** intestinal epithelial cells, occludin, paracellular transmigration, tight junction, *Toxoplasma gondii*

## Abstract

*Toxoplasma gondii* is a ubiquitous parasite and a prevalent food-borne parasitic pathogen. Infection of the host occurs principally through oral consumption of contaminated food and water with the gastrointestinal tract being the primary route for entry into the host. To promote infection, *T. gondii* has evolved highly specialized strategies for rapid traversal of the single cell thick intestinal epithelial barrier. Parasite transmigration via the paracellular pathway between adjacent cells enables parasite dissemination to secondary sites of infection where chronic infection of muscle and brain tissue is established. It has recently been proposed that parasite interactions with the integral tight junction (TJ) protein occludin influences parasite transmigration of the intestinal epithelium. We review here the emerging mechanisms of *T. gondii* transmigration of the small intestinal epithelium alongside the developing role played in modulating the wider TJ-associated proteome to rewire host cell regulatory systems for the benefit of the parasite.

## Introduction

*Toxoplasma gondii*, first isolated in 1908 from the African rodent *Ctenodactylus gundi*, is a protozoan obligate intracellular parasite of the phylum *Apicomplexa* that infects virtually all warm-blooded animals.^1^ It is recognized as a prevalent human pathogen, being one of the most significant sources of food-borne disease worldwide^2^ with infection commonly acquired by oral ingestion of undercooked meat or contaminated unwashed vegetables and water supplies.^3-6^ *T. gondii* infection is widespread throughout the world and in the United States and the United Kingdom it is estimated that 16–40% of the population have been infected by *T. gondii*, whereas in Central and South America and continental Europe, estimates of infection range from 50–80%.^7,8^ Although the incidence of *T. gondii* infection is high, with the exception of the immunocompromised and pregnant women, individuals usually present no signs of clinical infection other than mild flu-like symptoms with 80–90% of infections going unrecognised.^9^ *T. gondii* also has a considerable economic veterinary impact being a major cause of abortion and stillbirth in livestock.^10-12^

Following ingestion, *T. gondii* encounters the semi-permeable barrier of the single layer of intestinal epithelial cells,^13^ restricted by junctional complexes which occlude the paracellular space between neighboring cells.^14-16^ It is unclear currently, how *T. gondii* transmigrate the intestinal epithelium though it is essential for understanding disease pathogenesis. This review aims to build an understanding of how the success of *T. gondii* in global pathogenesis is linked with its ability to rapidly cross the epithelial barrier of the small intestine (SI), and to provide insights into how technological advances have enabled in-depth examination of how *T. gondii* interacts with the host cell, potentiating the design of novel intervention and protection strategies.

## Small intestinal epithelial barrier

The continuous ring of the epithelial junctional complex comprises 3 distinct morphological structures: the apical sealing TJ, adhesive adherens junction (AJ) and desmosomes that maintain cell-cell contacts.^17-19^ Apical TJs function both as a molecular “fence” dividing the epithelial cell plasma membrane into apical and basolateral domains and limiting the lateral diffusion of lipids and integral proteins between these domains and as a “gate” or barrier between the inside of the body and the external environment.^20^ This semi-permeable diffusion barrier permits the size and charge-selective transport of ions, solutes and water across the epithelium and is regulated by a continuous cycle of assembly and disassembly of the integral transmembrane proteins including occludin, junctional adhesion molecules (JAMs) and the claudin family members.

Now, 50 y since its discovery, the TJ is known to be more dynamic than the original perception as a static, rigid structure that simply sealed the paracellular space with considerable progress being made in the understanding of TJ structure, function and regulation. Dynamic interactions between the integral transmembrane proteins and the cytoplasmic plaque of peripheral adaptor, scaffold and signaling proteins also link the junctional membrane to the actomyosin cytoskeleton.^21^ Furthermore, bidirectional signaling to and from the cell interior regulates cellular differentiation, proliferation, migration and survival although the complex interplay between TJ molecular structure and function is only beginning to be understood.^22-26^ It is probable that the current list of known TJ components is incomplete as the molecular architecture of the TJ and functional interactions of the TJ proteins are still to be fully defined.

## *Toxoplasma gondii* host infection and dissemination

*T. gondii* has a complex life cycle involving a sexual life cycle that occurs only in the feline host with parasites ultimately being released as oocytes into the environment and an asexual lytic cycle of intracellular growth and multiplication within epithelial cells that occurs in all infected, intermediate hosts. Once ingested from the contaminated environment by intermediate hosts such as livestock, mice or humans the oocysts resist degradation in the stomach and eventually rupture and release bradyzoites into the intestinal lumen.^27^ After transmigration of SI epithelial cells, parasites convert into proliferative, motile tachyzoites, which undergo an asexual lytic cycle of intracellular growth and multiplication by endodyogeny before cell rupture and release of tachyzoites into both the intestinal lumen and underlying tissues of the lamina propria, activating an acute immune response.^28^ The immune response promotes conversion from motile tachyzoites to slow-replicating dormant bradyzoite cysts, which persist for the life-time of the host, usually without causing disease. In rare cases and in the event the host becomes immunocompromised, the cysts rupture causing encephalitis and in very rare cases, death of the host.^28,29^

The crucial step for *T. gondii* establishment of infection and subsequent parasite survival and proliferation is parasite attachment to, and transmigration of the intestinal epithelial barrier. The disease outcome of *T. gondii* infection is therefore highly dependent on parasite virulence, although surprisingly *T. gondii* population biology has identified a limited number of dominant strains. Serology samples from infected humans and domestic or farm animals from North America and Europe were used to group *T. gondii* serovars into clonal lineages I, II and III,^3031^ and the recently discovered haplotype 12.^32^ Although closely related, these clonal types possess different virulence both within and between host species.^33,34^ How these differences in parasite virulence are specifically linked to parasite transmigration of the host intestinal epithelium remains to be answered.

*In silico* approaches are being increasingly used to identify new genes of interest or genetic pathways. A recent study using whole genome sequencing of 62 strains of *T. gondii* found both large regions of conserved genes and specific regions showing enhanced variation. These regions were associated with secretory pathogenicity determinants (SPDs); genes encoding secretory proteins from micronemes (MICs), dense granules (GRA), rhoptries (ROPs) and the SAG-1-related sequence (SRS) superfamily that are associated with host transmission and infection. Highly diverse regions were particularly linked to GRA, ROP and SAG genes such as ROP17, ROP5, GRA3 and SAG3 and SAG2A, which have been previously implicated in murine virulence differences between strains. In contrast, parasite MICs which play a central role in cellular attachment by binding to host receptors, were found to be highly conserved, suggesting *T. gondii* strains may utilize a conserved repertoire of these host MIC receptors.^35^ Sequence variation in the conserved MIC16 gene for instance has been examined in 12 *T. gondii* strains covering the 3 major clonal lineages and all isolates could be grouped into their respective genotypes based on their MIC16 sequence.^36^ In addition, knockdown of surface antigen SAG3 resulted in 50% reduction in attachment in vitro and virulence in vivo.^37^

**Figure 1.**
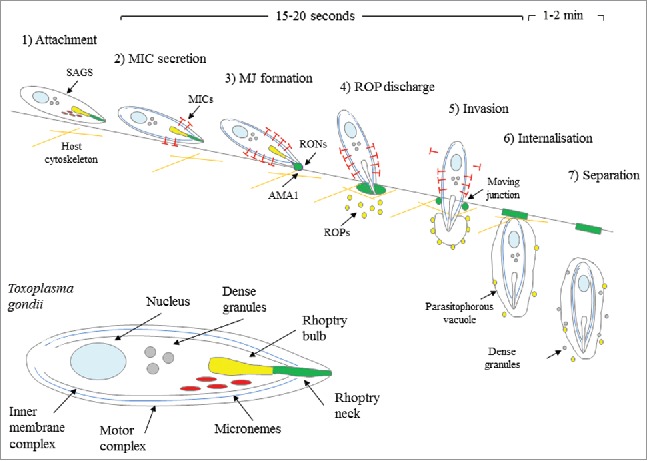
Strategies and timing of *T. gondii* transcellular invasion. (1) Initial attachment to the host cell surface via SAGs precedes (2) conoid extension, release of MICs and apical attachment. (3) Invasion is initiated by secretion of RONs and association with microneme-derived AMA1, which forms the ring-like MJ. (4) The parasite re-orientates and ROPs are discharged from rhoptries into the host cytoplasm where they associate with the developing PV or remain soluble. (5) The parasite actively invades through the MJ, creating the invaginated PV. (6) Once internalised, the PV is closed and 7) the parasite separates from the host plasma membrane and dense granules are released and associate with the PVM. Steps 2–5 take only 15–20 seconds whereas the final steps 6–7 take 1–2 minutes. Magnified view of tachyzoite (inset). Figure adapted from Carruthers and Boothroyd.^46^ © Elsevier. Reproduced by permission of Elsevier. Permission to reuse must be obtained from the rightsholder.

Parasite dissemination across distinct biologic barriers requires control of the diverse cohort of surface and secreted proteins. The transcellular or active penetration invasion mechanism has been well described for *T. gondii* tachyzoites, bradyzoites and sporozoites using various molecular and imaging-based techniques,^38^ summarised in Fig. 1. As *T. gondii* lacks cilia or flagella, the early stages of host cell entry involves an unusual form of gliding motility that was first observed over 100 y ago [For a review see^39^]. Initial contact with the host cell is then mediated by parasite surface molecules and complementary host cell ligands including parasite surface antigen-1 (SAG1) binding to host laminin via the β1 integrin receptor, first described over 20 y ago.^40,41^ Subsequently, differential SAG and SRS protein expression in *T. gondii* types I, II and II has been directly linked to parasite virulence.^42,43^ Although the functional significance of this correlation is currently unknown, evidence suggests *T. gondii* expression of surface antigens that demonstrate crossover functions or redundancy may account for the multitude of parasite-host interactions and broad parasite host range.

Following host cell surface attachment, the parasite undergoes a series of stages of reorientation and penetration depicted in Fig. 1 that are highly dependent on the differential expression of specialized polar organelles and inclusion bodies. These include the outer pellicle membranes, apical microtubule-containing conoid, secretory ROPs, MICs and GRAs and the plastid-like apicoplast.^44^ Apical conoid extension is followed by re-orientation, secretion of organelle contents and moving junction (MJ) formation between parasite surface proteins such as AMA1^45^ and the host cell membrane. The parasite then actively enters the cell through the MJ into the non-fusogenic parasitophorous vacuole (PV) using its actomyosin motor complex before separating from the host plasma membrane and initiation of replication.^46^ *T. gondii* does not actively invade all cells it attaches to. It has therefore been proposed that neighboring cells are prepared for a later wave of transmigration or invasion through organelle secretion of ROP and MIC proteins.^47-49^

Despite its clinical importance, relatively few drugs against *T. gondii* are currently available and only one vaccine, Toxovax is available for use in sheep and goats,^50^ but as it contains live attenuated tachyzoites, it cannot be used in humans. Potential new vaccines require careful selection of appropriate antigens to induce protective immunity, with surface or secreted antigens such as SAG, ROP, GRA and MIC associated with the initial stages of infection presenting promising candidates.^51^ Screening for drugs that effectively inhibit *T. gondii* attachment and invasion may also provide a starting point for the discovery of novel therapeutics; preliminary screening of a library of 1,120 compounds identified several encouraging targets. Pimozide, an inhibitor of dopamine signaling, for instance reduced parasite invasion by ∼50% but caution should be taken to rule out any off-target effects.^52^

## Transmigration of the small intestinal epithelium

Paracellular transmigration of the SI epithelial barrier presents a fast-track route of parasite dissemination to underlying tissues, avoiding replication and cytolysis that can lead to tissue injury and initiation of an acute immune response. This initial transmigration process is known to be rapid, taking only 20–40 seconds *in vitro*.^53,54^ with further dissemination to all organs of the body such as Peyers patches and lymph nodes within 2 d post infection (p.i), within the ilium within 5 d p.i, and to the brain and heart by 10 d p.i *in vivo*^55,56^

**Figure 2.**
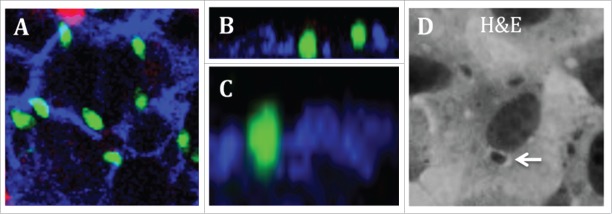
*Toxoplasma gondii* clustering to intestinal epithelial cell junctions. A-C) SI IEC-6 cells cultured on PET inserts were infected with *T. gondii* (green), fixed with PFA and stained for lateral membrane β-catenin (blue) and cell surface carbohydrates (red). (A) Image shows parasite clustering at cellular junctions. (B–C) Image shows parasites within the paracellular space between cells. (D) The parasitophorous vacuole is clearly visible as a white halo (white arrow) in cells cultured on glass coverslips after H&E staining.

**Figure 3.**
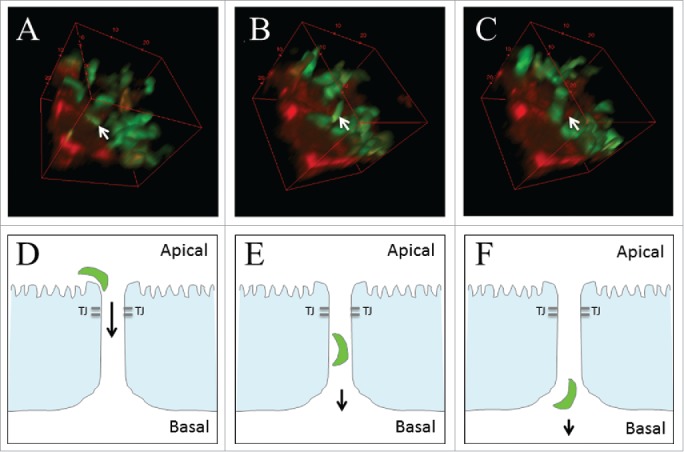
*Toxoplasma* paracellular infection. IEC-6 stained with CellTracker™ red (red) were apically infected with *T. gondii* (green) before 2-photon microscope live imaging. (A-C) 3D reconstruction of a transmigrating parasite (white arrow) targeting the epithelial cellular junction. Following initial localization to the cellular junction (A), the parasite re-orientates (B) and transmigrates the epithelium and disseminates from the SI (C). (D–F) Schematic representation of this proposed paracellular route of infection. Images are representative of those obtained from 2 experiments with replicates. Images were acquired using a LaVision BioTec TriM Scope II 2-photon microscope (Bielefeld) based on a Nikon Eclipse Ti optical inverted microscope. Z-stacks were separated by 1 μm. Images were analyzed with the Fiji/ImageJ package.

Exploitation of the paracellular pathway as a mechanism of infection and the role of TJ proteins, particularly the transmembrane proteins, in infection is not a new concept. In 2002, Barragan and Sibley first suggested this early wave of rapidly migrating parasites may be particularly important to ensure dissemination before activation of the immune system.^57^ These authors later described a significant proportion of tachyzoites clustering within 5μm of an intercellular junction during early infection and between host cells, adjacent to TJs. This implies parasites utilize the paracellular route to actively cross the epithelial barrier and avoiding damage to the epithelium and bypassing intracellular replication.^57^ Our own studies have confirmed the original observation made by Barragan and colleagues that tachyzoites cluster to cellular junctions (Fig. 2) and transmigrate through the SI epithelial barrier without altering barrier function. Fig. 3 shows *T. gondii* tachyzoites located in close contact with the apical cellular junction of cultured intestinal epithelial cells in the clear non-stained region between host cells. The parasite subsequently re-orientates and rapidly transmigrates through the paracellular space between host cells within 52 seconds, comparable with previous studies.

Following transmigration of the SI epithelium, *T. gondii* is known to exploit the motility of infiltrating SI immune cells in a Trojan Horse-like mechanism to spread throughout the body to secondary sites of infection, such as the muscle tissues or brain.^58,59^

We identified a role for intestinal epithelial lymphocytes (iIELs), which intimately associate with SI epithelial cells, in *T. gondii* infection.^60^ Increased susceptibility to infection and a striking *in vivo* change in occludin localization to the apical TJ was identified in mice lacking a specific γδT-cellpopulation of iIELs whereas in γδiIEL knockout mice no such redistribution occurred implying that γδiIEL interaction with SI epithelial cells played a central role in maintaining TJ integrity and barrier function in response to *T. gondii* infection.^60^ The leaky epithelium in γδiIEL-deficient mice was attributed to an absence of occludin phosphorylation and loss of claudin-3 and ZO-1 from the TJ complex. The possibility that *T. gondii* take advantage of a breach in the epithelial barrier provided in the absence of functional IELs was enhanced by a recent report using both γδT-cell deficient mice and those expressing migration-deficient, occludin-deficient, γδT-cells. Compared to WT mice, both KO models infected orally with *T. gondii* demonstrated increased translocation of parasites into the lamina propria.^61^

## The role of the tight junction in *t. gondii* transmigration

During natural infection the capacity for *T. gondii* to cross the barriers of the SI epithelium, placenta, blood-brain and blood-retina crucially enable parasites to breach host immune defenses and reach deeper muscle tissues. It is therefore somewhat surprising that little is known about the interactions between *T. gondii* and these host cellular junctions. In 2011, parasite infection of murine skeletal muscle cells (SkMC) demonstrated loss of the AJ protein cadherin and disruption of cell-cell contacts.^62^ Similarly, insights into the progression of ocular toxoplasmosis were recently provided by the discovery that parasite infection caused a breakdown in the retinal pigment epithelial cell (RPE) TJ complex with reduced TJ-associated occludin and increasing loss of cell-cell contacts with the progression of infection.^63^ Although these few studies provide limited insight into the effect of *T. gondii* on cellular junctions of various tissues, the role of the host TJ-associated network of proteins is still not well established.

As the first point of contact between parasite and host, the SI epithelial barrier has been more widely studied. We recently confirmed a role for TJ-associated occludin in the *T. gondii* paracellular route of infection using m-IC_c12_, an *in vitro* murine SI epithelial cell line.^64^ *T. gondii* tachyzoites were shown to both co-localize with TJ-associated occludin and cause re-distribution of occludin from an apical TJ location to the intracellular compartment; an effect more recently observed using the colorectal adenocarcinoma-derived Caco-2 cell line after 24 hours of infection.^65^ Using RNA interference (siRNA) to reduce endogenous occludin expression, we showed that parasite transepithelial migration was significantly attenuated, providing the first insight into occludin playing a key role in *T. gondii* paracellular transmigration of the SI epithelium.^66^

With the knowledge that occludin is crucial for transmigration, we also provided evidence for occludin acting as a receptor during parasite paracellular transmigration. Cell-free binding studies using recombinant TJ occludin proteins identified the potential interaction between *T. gondii* and the extracellular domains of occludin. This together with the recently captured high-resolution 2-photon live-cell imaging of *in vitro* paracellular transmigration of the SI epithelium, is consistent with *T. gondii* interacting directly with these domains to initiate rapid paracellular transmigration of SI epithelium.^66^

The ability of *T. gondii* to attach and invade almost any nucleated cell together with the expression of occludin in a broad range of cell types including endothelial cells of blood vessels and the blood-brain barrier,^67^ indicates this novel interaction between *T. gondii* and occludin may be a universal means of parasite paracellular transmigration of host tissue barriers including the SI epithelium. Further analysis of *T. gondii* binding to, and the effect on the junctional complex is required to reveal if drugs targeting this interaction could be a therapeutic option to block the early transmigration stage of infection and prevent toxoplasmosis.

## Modulation of tight junction-associated proteins

To investigate the involvement of host proteins during *T. gondii* transmigration of the SI epithelium several experimental approaches have been used. For instance immunoprecipitation with a soluble ICAM-1 antibody that identified *T. gondii* MIC2, a microneme adhesion protein discharged to the parasite surface and involved in parasite transmigration of the host epithelium.^68^ A number of groups have applied proteomics, the large-scale analysis of protein expression, to investigate host-parasite relationships. The entire *T. gondii* proteome^69-73^ or proteome sub-sets restricted to parasite excreted proteins^74^ or the rhoptry organelle have been defined which has identified changes in protein expression during *T. gondii* infection and novel potential host-parasite effector proteins including parasite-derived kinases, phosphatases and proteases.^75^ Host-cell proteins involved in modulation of a network of host processes such as the immune response, metabolism, cell cycle and apoptosis as well as cytoskeleton and organelle reorganisation have also been identified.^76-79^

It has to date been difficult to dissect the complex interactions between parasites and host cells as both share similar components such as actin cytoskeleton. However, recent advances in proteomics promise to provide new insights into the potential mechanisms of host cell subversion. Identifying components of the TJ protein complex modulated in response to *T. gondii* represents an important step toward understanding the molecular mechanisms of paracellular parasite infection and may aid the development of therapeutic strategies against *T. gondii*. However, this is complicated by an incomplete understanding of TJ composition and architecture, and by additional constituents yet to be identified.

Accordingly, we recently deployed a quantitative stable isotope labeling with amino acids in cells culture (SILAC) proteomics methodology to examine the small intestinal epithelial (IEC-6) cell response to *T. gondii* tachyzoite infection (unpublished observations). This methodology involves growing 2 populations of cells in culture media with either ‘light’ (normal) or ‘heavy’ (isotopically labeled) essential amino acids that are incorporated into all newly synthesized proteins. Here one cell population was infected with *T. gondii* and the other served as a non-infected control cell population. Post-infection, the 2 cell populations were mixed and the abundance of cellular proteins quantified by mass spectrometry. Using the known difference in molecular weight between ‘heavy’ and ‘light’ labeled peptides, termed the ‘mass shift’,^80^ enabled significant differences in IEC-6 host protein abundance due to parasite infection to be identified.

Subsequent comparison of the SILAC data set of host-derived proteins with a compiled TJ protein network, constructed by examination of current literature and integration of functional annotations, protein-protein interactions and known signaling pathways, identified 8 host TJ-associated proteins that significantly changed in abundance in response to parasite infection. Functional classification identified changes in catalytic, binding and structural proteins as well as statistically significant enrichment of differentially regulated proteins involved in cell metabolism, glycolysis, organelle organization, cellular transport, cell cycle, transcription, cell structure and the cellular stress response; analogous with findings from previous studies. For example within 24 hours of infection, host Bcar1 (Breast cancer anti-estrogen resistance-1, also known as p130CAS), Ybx3 (Y box binding protein 3, also known as ZONAB), Mras (Ras related protein) and Cstf2 (Cleavage stimulation factor 2, also known as Cstf-64) increased in abundance. Whereas Akt (RAC-α serine threonine protein kinase, also known as protein kinase B, PKB), Arhgef11 (Rho guanine nucleotide exchange factor (GEF) 11, also known as PDZ-RhoGEF), Cldn15 (Claudin-15) and Prkcι (Atypical protein kinase C ι, also known as aPKCι) all decreased in abundance.

**Figure 4.**
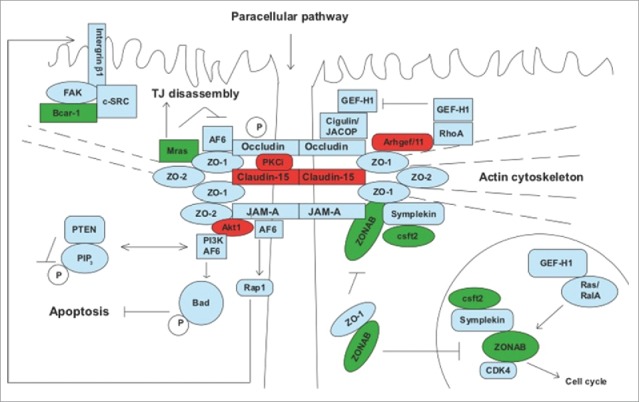
Modulation of the host TJ proteome during *T. gondii* infection. Schematic representation of the IEC-6 derived TJ-associated proteins modulated during infection and predicted downstream effector proteins and signaling pathways. Proteins demonstrating a significant increase in abundance are highlighted in red and a significant decrease in green.

By combining our current knowledge of how these proteins regulate the function of the epithelial TJ, either directly via integral transmembrane proteins or indirectly through their downstream network of cytoplasmic plaque, scaffolding, adaptor and signaling proteins enables the model shown in Fig. 4 to be generated. The wide range of proteins involved in these signaling networks often exhibit multiple roles and demonstrate extensive cross-talk, highlighting the striking connections between the *T. gondii* modulated TJ-associated proteins identified by the SILAC study. Identification of the newly discovered *T. gondii* MIC protein, claudin-like apicomplexan microneme protein (CLAMP), as showing structural similarity to claudins -15 and -19, is particularly intriguing as although the interaction between claudins and TJ transmembrane proteins such as occludin is not fully understood, claudins are known to interact in both cis and trans at the TJ and the occludin-claudin interaction may be important in regulation of the TJ barrier [For a review seeref.^81^]. Discovery of a claudin-like protein on the apical surface of *T. gondii* that shows intimate interaction with the host cell during infection therefore presents a promising candidate for interaction with host TJs during the initial stages of infection. Furthermore, disruption of the claudin-occludin complex may induce or inhibit various downstream signaling pathways such as programmed cell death, termed apoptosis. Primarily initiated by the host-cell to maintain the epithelial barrier by extruding damaged cells, parasite triggering of host-cell apoptosis may additionally allow parasite entry into the sub-epithelial compartment by creating gaps in the epithelial monolayer during acute infection. In contrast, the well-documented anti-apoptotic effect of intracellular, replicating parasites demonstrates the wide array of strategies evolved by *T. gondii* to manipulate the host cell and emphasizes the difficulties in unravelling the complex mechanisms of host-cell subversion.

Further work is now needed to provide functional studies corroborating these findings and answer a key question arising from this study; the degree of conservation of the hypothesized host-cell proteome response in the broad range of host tissue barriers targeted by *T. gondii*.

## Conclusions

*T. gondii* infection following consumption of contaminated food and water presents a significant worldwide health problem, potentiated by the parasites highly specialized strategies for rapidly crossing the barrier of intestinal epithelial cells that contributes to its success in infecting a wide range of hosts. Our understanding of *T. gondii* transmigration of the SI epithelium has been progressed by recent advances in high resolution microscopy and proteomics that have uncovered new questions that need to be answered to achieve a mechanistic model of *T. gondii* transmigration of the SI epithelium. Further in depth pathway reconstruction maps and network analysis of the proteins involved in signaling pathways downstream of the integral TJ proteins is required to fully understand interactions between the modulated host proteins and the impact of changes in abundance or redistribution. Modeling of the TJ signaling complex is challenging when considering TJ-associated protein-protein interactions may be weak or transient and TJ assembly and disassembly is highly dynamic occurring within seconds or minutes. Elucidating how the modulation of these signaling mechanisms contributes to *T. gondii* disease development may hold the key to developing successful future therapeutics.
